# A Reassessment of the *Marrubium Vulgare* L. Herb’s Potential Role in Diabetes Mellitus Type 2: First Results Guide the Investigation toward New Horizons

**DOI:** 10.3390/medicines4030057

**Published:** 2017-08-02

**Authors:** Javier Rodríguez Villanueva, Jorge Martín Esteban, Laura Rodríguez Villanueva

**Affiliations:** 1 Biomedical Sciences Department, Pharmacy and Pharmaceutical Technology Unit, Faculty of Pharmacy, University of Alcalá, Ctra. de Madrid-Barcelona (Autovía A2) Km. 33,600, 28805 Alcalá de Henares, Madrid, Spain; 2 Phytotherapy Faculty, University of Barcelona, Gran Via de les Corts Catalanes, 585, 08007 Barcelona, Spain; jorgemartinesteban@gmail.com; 3 Faculty of Pharmacy, University of Alcalá, Ctra. de Madrid-Barcelona (Autovía A2) Km. 33,600 28805 Alcalá de Henares, Madrid, Spain; lau95.rv@gmail.com

**Keywords:** *Marrubium vulgare* L. herb, diabetes mellitus type 2, clinical trial, methodology

## Abstract

Despite the wide variety of pharmacological activities described for the *Marrubium vulgare* L. herb, amazingly, only one clinical trial can be found in scientific literature. It was designed for the evaluation of its antidiabetic activity. Worse, the outcomes of this trial were contradictory to what previous in vivo mice assays had concluded. Therefore, should *Marrubium vulgare* be ruled out due to its lack of therapeutic potential in diabetes? The authors suggest a reevaluation of the clinical trial methodology to establish valid and final results.

## 1. Introduction

*Marrubium* L. (Lamiaceae) has about forty species, generally distributed in temperate regions of Central and Western Asia, North Africa, Europe, and South America. Generally, these plants are large annual or perennial shrubs [1]. The furane labdane diterpene marrubiin is assumed to be the chemotaxonomic marker among the various species of the *Marrubium* genus. Generally, marrubiin is isolated from dried whole plants of *Marrubium vulgare* Linn (white/common horehound, hoarhound, marriout, maromba, marroio-branco, or marrubio) in a proportion of 0.3–0.7% [2]. *M. vulgare* L., commonly known as “white horehound” can be identified as a robust perennial herb, with densely cottony stems and white flowers [3].

**Figure 1:**
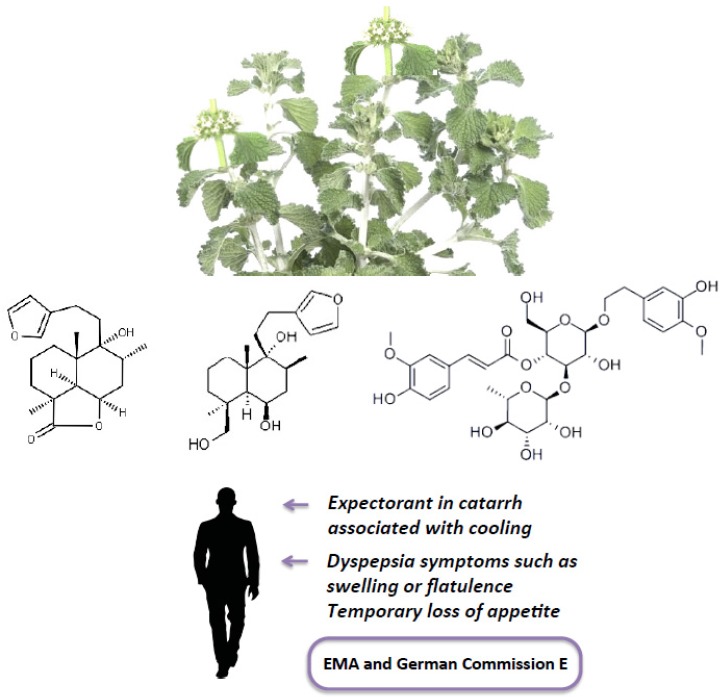
Top, photograph of the aerial part of *Marrubium vulgare* L.; Middle, marrubiin, marrubiol (both diterpenes), and martinoside (a phenylpropanoid), three of the plant’s active compounds; Bottom, the European Medicine Agency (EMA) and German Commission E approved indications for the aerial part of *Marrubium vulgare* L.

Researchers have tried to support the biological activities described for the *M. vulgare* L. herb (for an extended analysis, see Reference [4] and Figure 1) by the isolation and identification of biologically active phytochemicals such as diterpenes (marrubiin and related compounds), flavonoids (luteolin, apigenin, ladanein, quercetin, isoquercitrin, chrysoeriol, or vitexin), phenylpropanoid esters (including acteoside (or verbascoside), forsythoside B, arenarioside, ballotetroside, alyssonoide, marruboside, and acethyl marruboside), tannins (such as proanthocyanidins, catechin and epicatechin, condensed tannins), and sterols [5].

Diabetes mellitus is a metabolic disease characterized by chronic hyperglycemia resulting from defects in insulin secretion, insulin action, or both. Diabetes mellitus type 2 (DM2), known as adult type or non-insulin-dependent diabetes mellitus, is treated by controlling the diet and oral hypoglycemic drugs [6]. In 2004, more than 120 million people in the world had diabetes. In 2015, there were approximately 415 million people with diabetes, and this is projected to increase to 642 million by 2040 (International Diabetes Federation atlas, 2015). In Mexico, DM2 affects 8.2% of the population between 20 and 69 years old and has the highest mortality rate of chronic degenerative diseases, representing 16.7% of deaths [7].

*M. Vulgar*e L. has been reported to be used in the treatment of diabetes in traditional medicine in Mexico [8] and central Morocco [6], and scientific studies have revealed through in vivo research the hypoglycemic effect of this plant, supporting its traditional use in diabetes mellitus control [9].

## 2. Preclinical In Vivo Observations: The Starting Point

Initial studies showed that horehound infusion (132 g of the dried plant/1 L water, in a dose of 4 mL/kg body weight) decreased the glucose curve significantly when a 50% dextrose solution (4 mL/kg of weight) was administered [10].

Years later, the methanolic extract of the top parts of *M. vulgare* L. was also evaluated for the same purpose [11], and 500 g of the aerial part was homogenized with methanol for 15 min, three times each with 1000 mL (500 g/1 × 3 L), followed by distillation of the solvent under reduced pressure. The percentage yield was calculated as 12%. In this case, diabetes was induced in rats through streptozotocin (in 0.1 M sodium citrate buffer, pH 4.5) injected intraperitoneally at a dose of 55 mg/kg, as a single dose. The diabetic rats orally received the extract of *M. vulgare* in a dose of 500 mg dry extract/kg of weight in a 1% CMC-Na vehicle once a day, starting on the 11th day. The oral administration of the *M. vulgare* extract significantly reduced the plasma glucose level after three days by more than 7% (curiously, glibenclamide has no effect here). After 14 days, the reduction was marked, reaching 42% compared to the control values of the diabetic group. The plasma glucose level was reduced in the glibenclamide group by 31% compared to the control values of the diabetic group on the 28th day.

In 2012, Boudjelal et al. [9] proved through preclinical in vivo trials the hypoglycemic and hypolipidemic effects in diabetic albino rats (180–200 g) fed ad libitum with a pellet diet and water, kept and maintained under laboratory conditions of temperature and light (24 ± 1 °C and a 12 h light/dark cycle). Rats were randomly divided into six groups and injected intraperitoneally with a single injection of alloxan monohydrate (150 mg/kg) to cause hyperglycemia and fasting blood glucose levels greater than 300 mg/dL.

The extract of *M. Vulgare* administered was prepared by boiling 6 g of the aerial parts of the plant, dried at room temperature in the dark and ground to a powder, in 25 mL of distilled water for 15 min, the mixture of which was then left to reach room temperature and filtrated. Extracts in a dose of 100, 200, or 300 mg/kg of body weight were orally administered twice daily for 15 days. The positive control was glibenclamide (5 mg/kg of body weight); a normal control and a diabetic control were also included. A sharp decline in blood glucose levels was observed from the third day after the treatment with three doses of *M. vulgare* extracts and glibenclamide (more than 5% for 300 mg/kg of body weight, and up to 12% for glibenclamide). In particular, the highest percentage decrease of glycaemia levels was observed after 28 days for the treatments with 300 mg/kg of body weight of *Marrubium* infusion (−62.55%) and the positive control glibenclamide (−65.90%). Serum glucose, total lipids, triglycerides, and total cholesterol decreased after the administration of *M. vulgare* extract in all doses without attaining the values of the normal control. The effect was similar to that observed with the positive control, glibenclamide. The authors attribute these results to a stimulation of insulin secretion from beta cells of islets and/or inhibition of insulin degradation processes due to the high content of flavonoids in the drug (15.53 mg quercetin equivalent/g of dry plant material).

In both studies, there were no physical signs of toxicity, such as writhing, gasping, palpitation and respiratory rate, or mortality in the rats. The rats treated with different doses of *M. vulgare* did not show any drug-induced behavioral disorders.

## 3. From Animals to Humans: The Clinical Trial Issue for Herbal Medicine

More than 10 years ago, Wolsko and coworkers [12] stated that poor quality control in the United States was not surprising, given the US regulatory environment as dictated by the Dietary Supplement Health and Education Act of 1994. The same could be applied nowadays, for the United States as well as for the European Union. There is still currently no minimum standard of practice for manufacturing dietary supplements, no premarket safety or efficacy studies are needed, and dietary supplements do not need approval from the Food and Drug Administration (FDA) or the European Medicine Agency (EMA) before they are marketed. It continues to be purely the manufacturers’ responsibility to ensure that supplements are safe and labeled properly before marketing. The FDA or the EMA (coordinated with the agency of each member state) can take action to restrict a product’s use only after it has been shown that a dietary supplement is unsafe, sometimes years later, when it has taken the lives or has cost the health of innocent people. In the absence of evidence to the contrary, herbal supplements used in clinical trials have the same poor quality as demonstrated in the marketplace as a whole. This fact, linked with the number of pharmacognosy drugs of the same species and the different treatments being undergone, makes it very difficult if not impossible to establish valid conclusions that are not contradictory to what has been reported before in animals.

For the *Marrubium vulgare* L. herb, while some articles describe and sustain the ethnopharmacological use [13], only one clinical trial can be found in the scientific literature up to date [7]. This is striking given that, in 2015, preparations of this species were the best-selling herbal dietary supplements, reaching approximately $106 million in retail sales [14]. This randomized, double-blind, and controlled clinical trial was conducted to evaluate the clinical effect produced by its aqueous extract on type 2 non-controlled diabetes mellitus. The verum product consisted of fresh *M. vulgare* L. leaves that were dried under environmental temperatures and protected from direct light and then milled. Ethylene oxide was used for sterilization. Patients had to prepare the treatment immediately before administration. A 1-g filter-paper envelope was placed in a cup of boiling water for 5 min. *M. vulgare* L. extract was administered three times a day, before every meal. The infusion was analyzed through HPLC with UV detection at 255 nm only for chlorogenic acid determination. This compound was not found in the extract.

Outpatients of either sex, between 30 and 60 years old, who had been diagnosed with type 2 diabetes no more than five years earlier, were selected. All patients were under medical treatment but showing a fasting blood glucose >140 mg/dL. As usual, patients affected with diabetes complications or nephropathy, pregnant women, patients with gestational diabetes, and hospitalized patients were not selected. In this study, insulin-dependent or type I diabetics were excluded, and, for comfort and to assure results, people who needed to travel frequently were also not selected. A total of 43 patients were recruited, and 21 received an *M. vulgare* L. infusion. The other 22 received a *Cecropia obtusifolia* extract. All patients showed treatment adherence, evaluated by counting the used dosages.

The study was carried out for 21 days. Prior to infusion administration, every seven days and after the clinical trial, the fasting determination of glucose, cholesterol, triglycerides, urea, creatinine, and uric acid in blood was carried out using automatic equipment (Autolab) with standardized techniques by a certified external laboratory.

In this study, effectiveness was considered a decrease in the basal concentration of glucose, cholesterol, or triglycerides by at least 25%. *M. vulgare* L. caused that effect in only two of the 21 patients (9.52%). The mean of plasma glucose level was reduced by 0.64%, and that of cholesterol and triglycerides by 4.16% and 5.78%, respectively. These results were disappointing.

## 4. Some Result Considerations Must Be Kept in Mind: Paving the Way for Future Actions

Nevertheless, some considerations must be taken into account. First of all, as the authors state, a crude extract was evaluated, with a similar preparation to that used in traditional medicine. As previously described, effective results on animal models were obtained after the administration of specific extracts, available after processing crude plant material with different methods. The effect of preparation methods on composition or content (as well as on undesired ingredients) is not fully understood. The same applies for therapeutic activity. Also, the administration route influences the bioavailability, pharmacological activity, and clinical effectiveness of a phytotherapeutic preparation. Previously, clinical studies could only demonstrate observations if the botanic drug material and the study design were rationally based on that basis.

What is important here is that 6-octadecynoic acid, a fatty acid with a triple bond found in *Marrubium vulgare* L., exhibits PPARγ agonist activity by directly binding to helix 12 through the conformational change of the Ω loop. Compounds with PPARγ agonist activity have been clinically used for treatment of type 2 diabetes by improving insulin resistance [15]. However, it is only found in the organic (methanolic) extract of the aerial parts (tops, leaves, and flowers), as fatty acids are not soluble in water infusion. It is not difficult to propose that, between others, there may be a presumable relation between the efficacy of the methanolic extract in mice models/lack of efficacy as an aqueous extraction and the presence/absence of 6-octadecynoic acid.

Despite this, as we previously stated [16], a characterized compound with a perfectly defined mechanism of action at a particular dose fulfills the negative requirements (such as toxicity) inherited from classical pharmacology. However, new trends suggest an integrative approach in which a wide variety of compounds act on multiple targets together to produce a final action through a balance resulting from minor changes (synergy); something different is not expected here. Diterpenes, flavonoids, and phenylpropanoid esters may also play a role in the antidiabetic activity or in the diabetes concomitant effects as on animal models.

No less important is a diligent analysis of the dose translation from animals to humans, if possible with recognized recommendations (i.e., FDA, EMA, or Commission E if available). Elberry and colleagues [11] administered to rats 500 mg of dried extract (as previously described) per kg of weight. For an average person (weighing 60 kg), this dosage correlates with a dose of 81 mg/kg of weight. Taking into account the drug/extraction ratio, almost 39 g of the herbal substance (crude drug) is needed, which is impossible to achieve for an individual administration (the EMA recommends a 1–2 g dose of the cut drug, and the German Commission E recommends a 4–5 g dose of the fresh/dried plant material per day). Considering the hypothesis that 6-octadecynoic acid is the compound primarily involved in the antidiabetic activity and available in sufficient amounts of raw material, these results reveal that a more efficient extraction method should be developed (ethyl acetate showed good results in the study by Ohtera et al. [15], but possible toxicity risks must be abolished before achieving clinical trials with EtOAc extracts).

For example, we proposed that the extract prepared by El Bardai et al. [17,18] was obtained by aqueous infusion (5 g air parts/100 mL of water, yield after lyophilization) with a drug: extract ratio (DER) 6.2:1 and performance 13%. This extract minimizes possible toxicity due to organic solvents, allows oral administration, and makes feasible the fulfilment of the recommended therapeutic range, which allows a maximum amount of 6 g per drug per day.

Finally, it is important to notice the slighter side effects (described by one in every four patients) in the clinical trial, including nausea, oral dryness, sialorrhea, dizziness, and anorexia, although they were not adverse enough to necessarily cause a withdrawal from the study [7]. These results and the acquired experience in the traditional intake of the water infusion of *Marrubium vulgare* L. can be considered safe. In spite of this, the number of patients, as is usual in clinical trials of herb preparations, was reduced, and the establishment of general conclusions is therefore almost impossible.
